# Is a Genome a Codeword of an Error-Correcting Code?

**DOI:** 10.1371/journal.pone.0036644

**Published:** 2012-05-23

**Authors:** Luzinete C. B. Faria, Andréa S. L. Rocha, João H. Kleinschmidt, Márcio C. Silva-Filho, Edson Bim, Roberto H. Herai, Michel E. B. Yamagishi, Reginaldo Palazzo

**Affiliations:** 1 Departamento de Telemática, Universidade Estadual de Campinas, Campinas, São Paulo, Brazil; 2 Centro de Engenharia, Modelagem e Ciências Sociais Aplicadas, Universidade Federal do ABC, Santo André, São Paulo, Brazil; 3 Departamento de Genética, Escola Superior de Agricultura Luiz de Queiroz, Universidade de São Paulo, São Paulo, Brazil; 4 Departamento de Sistema de Controle de Energia, Universidade Estadual de Campinas, Campinas, São Paulo, Brazil; 5 Department of Cellular & Molecular Medicine, School of Medicine, University of California San Diego, La Jolla, California, United States of America; 6 Embrapa Informática Agropecuária, Laboratório de Bioinformática Aplicada, Campinas, São Paulo, Brazil; Pennsylvania State University, United States of America

## Abstract

Since a genome is a discrete sequence, the elements of which belong to a set of four letters, the question as to whether or not there is an error-correcting code underlying DNA sequences is unavoidable. The most common approach to answering this question is to propose a methodology to verify the existence of such a code. However, none of the methodologies proposed so far, although quite clever, has achieved that goal. In a recent work, we showed that DNA sequences can be identified as codewords in a class of cyclic error-correcting codes known as Hamming codes. In this paper, we show that a complete intron-exon gene, and even a plasmid genome, can be identified as a Hamming code codeword as well. Although this does not constitute a definitive proof that there is an error-correcting code underlying DNA sequences, it is the first evidence in this direction.

## Introduction

Frequently in science, two seemingly unrelated fields find common ground in a research problem of interest. For example, the fields of biology and coding theory share the same challenge, which is to answer the question of whether or not there is an error-control mechanism in DNA sequences similar to the one employed in digital transmission systems. There are several facts about DNA sequences which motivate this line of questioning. One is that DNA sequences may be viewed as “words” written using four letters or nucleotide bases. Another is that some DNA patches code for protein sequences. Furthermore, several DNA sites have been well annotated in terms of pattern and information content [1]. The evolution of these biologically significant sequences is usually evolutionarily conserved, and it is important to avoid sequence errors in order to maintain their function. Another interesting point is that the number of genes an organism has does not correlate with its complexity. In fact, the number of non-coding DNA (ncDNA) regions, including repetitive sequences, seems to have been increasing since the beginning of the evolution of the higher eukaryotes, which suggests that organism complexity is related to gene regulation through ncDNA [2]. It is well established that non-coding sequences are biologically important; e.g. regulatory regions (promoters, TFBS, enhancer elements, ncRNA, introns, splicing sites etc). Finally, and most importantly, the DNA replication process is far from being the only source of sequence errors. DNA integrity is frequently jeopardized by physical and chemical agents, which means that DNA damage repair mechanisms are indispensable in preventing collateral effects [3]. Interestingly, more than one of these mechanisms is described in the literature [4]. Is it reasonable to infer that some DNA repair mechanisms are a biological implementation of error-correcting codes?

The coding theory community has proposed several methodologies to verify whether or not a particular DNA sequence, usually a protein coding sequence, has an underlying error-correcting code (ECC) [5] and [6]. In spite of their relevance, the results of earlier works do not provide the definitive answer. For instance, based on the procedure for determining whether or not the *lac operon* and *cytochrome c gene* can be identified as codewords of linear block codes, the answer is no [7]. Actually, we cannot even conclude that there is no linear block code in other DNA sequences.

Of course, as is often the case, there is at least one alternative approach to solving this problem, which is to demonstrate that an ECC underlies DNA sequences. This task is far from easy to accomplish, because a complex error-correcting scheme might consist of many distinct concatenated codes, rather than a single global one, although, to the best of our knowledge, there is no evidence that such an ECC exists. In [8], we attempted to answer a recurring question: Are there DNA sequences that can be identified as codewords for ECCs? If so, we will have taken the first step in a long research journey. The majority of candidate DNA sequences have been positively identified as codewords for a class of cyclic block codes. Such codewords are consistently different from actual DNA sequences by one single nucleotide. Is this difference biologically significant? Are these codewords actually ancient DNA sequences? Up to now, researchers in the fields of biology and coding theory have been working almost independently of one another, and the two groups need to work together to address the new challenges. In this paper, we ask whether or not a whole intron-exon gene structure can be identified as a codeword, and, furthermore, can a whole genome be identified as a codeword? In the following sections, we describe our experiments and results.

## Methods

### BCH Code

ECCs are always used when transmitting or storing information. The main objective of an ECC is, as the name suggests, to correct errors that might occur during information transmission through noisy channels. BCH codes form a subset of parameterized ECCs, which were first proposed in 1959 by Hocquenghem [9] and independently rediscovered by Bose and Chaudhuri [10] in 1960. The acronym BCH is made up of the initials of Bose, Chaudhuri, and Hocquenghem, in that order. Usually BCH codes are employed in the transmission of information in computer networks and in sequence generation. Due to the simplicity of their encoding and decoding processes, these codes are good candidates for use in the identification and reproduction of DNA sequences, [8], [11]–[19]. By “identification”, we mean that the DNA sequence may be either a codeword for an ECC or one of the code sequences. These code sequences may differ from the codeword up to the error correction capability of the code. In the latter case, we say that such code sequences belong to a codeword set. The BCH codes constitute an important generalization of the Hamming codes by allowing multiple error corrections. The parameters associated with a BCH code are denoted by 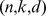, where 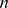 is the codeword length (number of base pairs in DNA sequences); 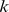 is the code dimension (length of the input information sequence responsible for generating the DNA sequence); and 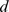 is the minimum code distance (the smallest number of positions by which any two codewords may differ).

### Converting Nucleotides into Numbers

**Figure 1:**
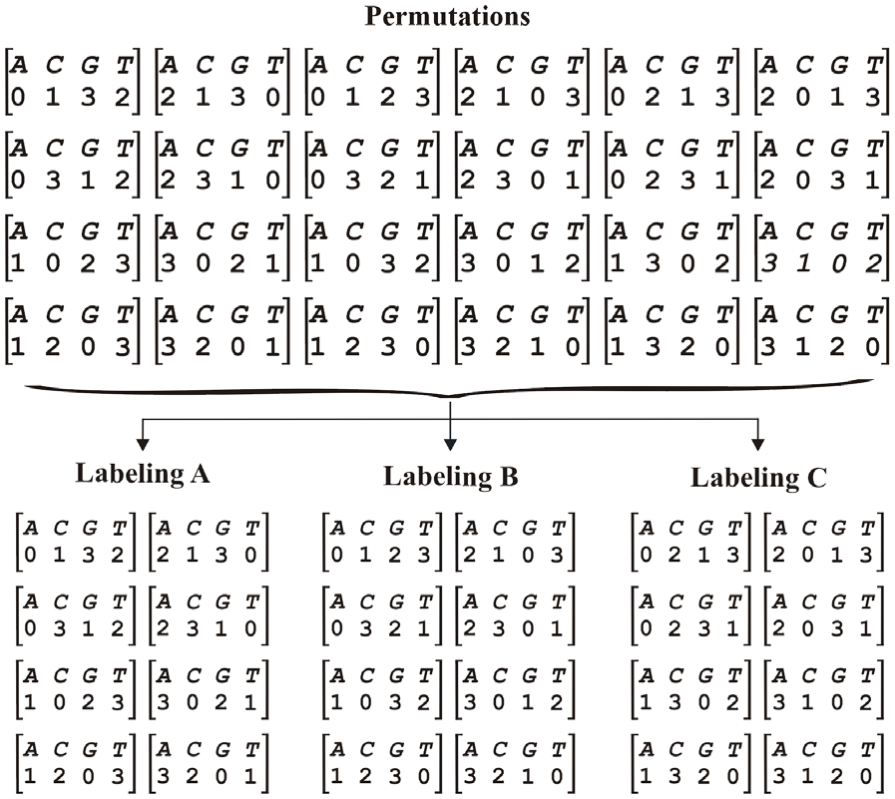
Permutations associated with labelings 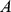, 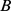 and 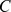.

It is desirable that the alphabet of an ECC have an associated algebraic structure. Although the genetic code has an associated alphabet, the identification of a related algebraic structure remains an open problem. We have considered the ring of integers modulo 4, denoted by 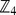, owing to the easy of code construction of using this algebraic structure. Since the alphabet of the genetic code must be converted into the alphabet of the ECC, and vice-versa, it follows that this conversion has to take into consideration all the possibilities of associating the elements of the set 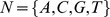, where 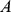 is adenine, 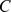 is cytosine, 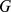 is guanine, and 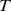 is thymine, with the elements of the set 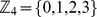. We call this association a labeling. The labeling between the set of nucleotides 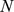 and the set 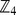 consists of the twenty-four permutations involved, as shown in Figure 1. The aim of these labelings is to determine which permutation matches the codeword with the given DNA sequence.

Next, in order to match the length of the DNA sequence to the codeword length, we must find the degree of the Galois ring extension, denoted by 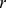, using the equality 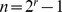, where 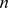 is the DNA sequence length in base pairs. For instance, if 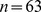, then the degree of the Galois ring extension 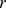 is 6. The primitive polynomial is obtained once we know the value of 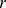, and, for every value of 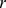 there are many primitive polynomials to consider. In looking for a new code, we have observed that there is a generator polynomial 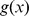 of the BCH code that corresponds to each primitive polynomial 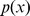.

In the code construction process, the DNA sequence generation algorithm takes into consideration three important facts. The first is to consider every possible value taken by the minimum distance 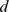 of the code, that is, 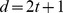, where 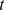 denotes the number of errors the code is able to correct. The second is to consider all 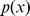 with degree 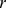 to be used in the Galois ring extension, 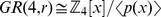 (Step 2 and Step 3) and all labeling A, B and C (Step 4), owing to the as yet unknown interdependence of the geometric and algebraic structures in the code construction, where 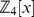 denotes the ring of all the polynomials with coefficients in 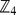, and 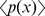 denotes the ideal generated by 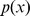. The third is to consider determining the group of units 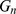 in 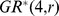, where 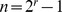 denotes the cardinality of 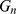 and 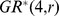 denotes the set of all non zero elements in 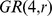. The additional computational complexity in the solution of this problem comes from the fact that the greater the degree of the Galois ring extension, the larger the number of 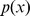 to be considered in the code construction.

Knowing that the number of codewords generated by these codes grows exponentially with the code dimension, instead of generating all the codewords and comparing them with the given DNA sequence, the twenty-four permutations are applied to that DNA sequence, and these sequences are considered as “possible codewords”. Then, to determine which of the twenty-four sequences are, in fact, codewords, the relation 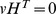 is employed, where 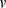 is each of the possible codewords and 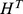 denotes the transpose of the parity-check matrix. The analysis to be performed with the DNA sequence, as a result of the one nucleotide difference from the codeword, is to consider the other three possible nucleotides at each position in the sequence for each permutation, and again to use the relation 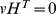, in order to verify whether or not 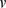 is a possible codeword.

Single stranded DNA sequences, such as single stranded chromosomes, genes, introns, exons, repetitive DNA, and mRNA sequences, may be either a codeword for an ECC or belong to the codeword set of an ECC. In order to verify whether or not a DNA sequence may actually be identified as a codeword, we can use an ad hoc strategy, i.e. generate all the codewords and compare the DNA sequence with each codeword. However, this is not a practical strategy, because the computational effort to do this would be prohibitive, as explained below. In order to address this identification problem, we have developed an algorithm called the DNA Sequence Generation Algorithm, which verifies whether or not a given DNA sequence can be identified as a codeword of an ECC. This algorithm is the same as the one in [8], however it differs from the algorithm in [20] in that it considers the Galois ring extension as the algebraic structure, instead of the Galois field extension. There are also some conceptual differences, which are discussed in [15] and [17].

### DNA Sequence Generation Algorithm

Input data: 1) 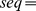 original DNA sequence in nucleotides (*NCBI*); 2) 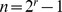; and 3) 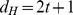.

- **Step 1** - Generate all primitive polynomials 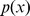 with degree 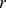 to be used in the Galois ring extensions;
- **Step 2** - Select one 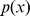 from **Step 1**, and find the set in which the elements have the inverse, the group of units of 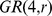, denoted by 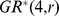;
- **Step 3** - Find the generator and parity-check polynomials of the BCH code by knowing the minimum distance and the primitive polynomial derived in **Step 2**. In this way, the generator, as well as the parity-check matrices and its transposes, are determined;
- **Step 4** - From the mapping 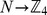, convert the *seq* with elements in 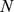 into the corresponding sequence with elements in 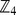;
- **Step 5** - Verify by use of the syndrome 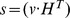, whether or not each of the converted DNA sequences is a codeword:
  If 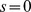, then store the sequence;
  If 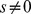 implies that up to 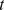 nucleotide differences may exist. If so, then the 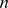 combinations 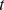 to 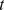 must be considered by taking into account the other three nucleotide possibilities in each of the combinations of the DNA sequence. Verify that every combination is a codeword: if so, store it; otherwise disregard it;
- **Step 6** - From the mapping 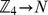 convert each stored sequence in **Step 5** with elements in 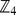 into the corresponding sequence with elements in 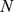. Compare each of these sequences with the *seq* and show the position at which the nucleotides differ;
- **Step 7** - Go to **Step 1**. Select another 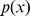 and verify whether or not all the 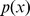 have already been used: if not, repeat **Steps 2** to **6** for each 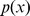 from **Step 1**; otherwise, go to **Step 8**.
- **Step 8** - End.

## Results and Discussion

We have successfully applied this algorithm to the TRAV7 gene sequence and the plasmid *Lactococcus lactis* genome sequence. These sequences are represented in Table 1 and Table 2 using the following abbreviations: Ont = original nucleotide; Olb = original labeling; Glb = generated labeling and Gnt = generated nucleotide. Although we have used all the 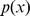, all the corresponding 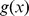, and all the possible minimum code distances in the construction of the BCH code over 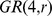, the results show that only codes with the minimum distance 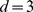 associated with a specific 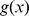, which in turn is associated with its 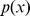 and labeling, are able to identify the TRAV7 gene and the plasmid genome sequences. Consequently, the algebraic structure, alphabet, labeling, 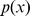, and 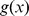 have to be considered in the construction of BCH codes over rings.

**Table 1:** *TRAV7 gene sequence* chromosome 14.

1	Ont:	atggagaaga	tgcggagacc	tgtcctaatt	atattttgtc	tatgtcttgg	ctgtaagttg
	Olb:	0311010010	3121101022	3132230033	0303333132	3031323311	2313001331
	Glb:	0311010010	3121101022	3132230033	0303333132	3031323311	2313001331
	Gnt:	atggagaaga	tgcggagacc	tgtcctaatt	atattttgtc	tatgtcttgg	ctgtaagttg
61	Ont:	agggttctaa	gaactgggga	ccccaggaga	catttattca	agtccttttg	gggagatggg
	Olb:	0111332300	1002311110	2222011010	2033303320	0132233331	1110103111
	Glb:	0111332300	1002311110	2222011010	2033303320	0132233331	1110103111
	Gnt:	agggttctaa	gaactgggga	ccccaggaga	catttattca	agtccttttg	gggagatggg
121	Ont:	gatgtagtct	ggacttactt	gtcattgctt	gtttgagatt	aagaaataaa	attatgaaag
	Olb:	1031301323	1102330233	1320331233	1333101033	0010003000	0330310001
	Glb:	1131301323	1102330233	1320331233	1333101033	0010003000	0330310001
	Gnt:	ggtgtagtct	ggacttactt	gtcattgctt	gtttgagatt	aagaaataaa	attatgaaag
181	Ont:	gtctaaatta	aaatgtacat	attgtacctg	atgtctttct	gaataggggc	aaatggagaa
	Olb:	1323000330	0003130203	0331302231	0313233323	1003011112	0003110100
	Glb:	1323000330	0003130203	0331302231	0313233323	1003011112	0003110100
	Gnt:	gtctaaatta	aaatgtacat	attgtacctg	atgtctttct	gaataggggc	aaatggagaa
241	Ont:	aaccaggtgg	agcacagccc	tcattttctg	ggaccccagc	agggagacgt	tgcctccatg
	Olb:	0022011311	0120201222	3203333231	1102222012	0111010213	3122322031
	Glb:	0022011311	0120201222	3203333231	1102222012	0111010213	3122322031
	Gnt:	aaccaggtgg	agcacagccc	tcattttctg	ggaccccagc	agggagacgt	tgcctccatg
301	Ont:	agctgcacgt	actctgtcag	tcgttttaac	aatttgcagt	ggtacaggca	aaatacaggg
	Olb:	0123120213	0232313201	3213333002	0033312013	1130201120	0003020111
	Glb:	0123120213	0232313201	3213333002	0033312013	1130201120	0003020111
	Gnt:	agctgcacgt	actctgtcag	tcgttttaac	aatttgcagt	ggtacaggca	aaatacaggg
361	Ont:	atgggtccca	aacacctatt	atccatgtat	tcagctggat	atgagaagca	gaaaggaaga
	Olb:	0311132220	0020223033	0322031303	3201231103	0310100120	1000110010
	Glb:	0311132220	0020223033	0322031303	3201231103	0310100120	1000110010
	Gnt:	atgggtccca	aacacctatt	atccatgtat	tcagctggat	atgagaagca	gaaaggaaga
421	Ont:	ctaaatgcta	cattactgaa	gaatggaagc	agcttgtaca	ttacagccgt	gcagcctgaa
	Olb:	2300031230	2033023100	1003110012	0123313020	3302012213	1201223100
	Glb:	2300031230	2033023100	1003110012	0123313020	3302012213	1201223100
	Gnt:	ctaaatgcta	cattactgaa	gaatggaagc	agcttgtaca	ttacagccgt	gcagcctgaa
481	Ont:	gattcagcca	cctatttctg	tgctgtagat	g		
	Olb:	1033201220	2230333231	3123130103	1		
	Glb:	1033201220	2230333231	3123130103	1		
	Gnt:	gattcagcca	cctatttctg	tgctgtagat	g		

**Table 2:** *Lactococcus lactis* plasmid genomic sequence.

1	Ont:	cctacatttt	tttattgctc	tgctatgatt	gtttatcgat	agttttttat	acagataagc
	Olb:	1130103333	3330332131	3213032033	2333031203	0233333303	0102030021
	Glb:	1130103333	3330332131	3213032033	2333031203	0233333303	0102030021
	Gnt:	cctacatttt	tttattgctc	tgctatgatt	gtttatcgat	agttttttat	acagataagc
61	Ont:	gtgcgacgct	tgctctttcc	gaggaggaag	tcatgctgac	aagcacggca	gagcctccgc
	Olb:	2321201213	3213133311	2022022002	3103213201	0021012210	2021131121
	Glb:	2321201213	3213133311	2022022002	3103213201	0021012210	2021131121
	Gnt:	gtgcgacgct	tgctctttcc	gaggaggaag	tcatgctgac	aagcacggca	gagcctccgc
121	Ont:	atgaaatgct	ctcaatgaaa	ttgccggcgg	agcttttttg	agcttgtgcc	acttgcgaaa
	Olb:	0320003213	1310032000	3321122122	0213333332	0213323211	0133212000
	Glb:	0320003213	1310032000	3321122122	0213333332	0213323211	0133212000
	Gnt:	atgaaatgct	ctcaatgaaa	ttgccggcgg	agcttttttg	agcttgtgcc	acttgcgaaa
181	Ont:	aaaacaagaa	caaaagagac	aggaaactgt	ctttttttgc	ttgcttgggg	attggggcaa
	Olb:	0000100200	1000020201	0220001323	1333333321	3321332222	0332222100
	Glb:	0000100200	1000020201	0220001323	1333333321	3321332222	0332222100
	Gnt:	aaaacaagaa	caaaagagac	aggaaactgt	ctttttttgc	ttgcttgggg	attggggcaa
241	Ont:	cgccccaaaa	ataaaaagaa	tcgtctgaaa	cgaggaacaa	actaaaatgt	aaattttagt
	Olb:	1211110000	0300000200	3123132000	1202200100	0130000323	0003333023
	Glb:	1211110000	0300000200	3123132000	1202200100	0130000323	0003333023
	Gnt:	cgccccaaaa	ataaaaagaa	tcgtctgaaa	cgaggaacaa	actaaaatgt	aaattttagt
301	Ont:	tgttaccgag	tggaagatga	atacttttta	acctatgtgt	atacacacat	agtaagctcg
	Olb:	3233011202	3220020320	0301333330	0113032323	0301010103	0230021312
	Glb:	3233011202	3220020320	0301333330	0113032323	0301010103	0230021312
	Gnt:	tgttaccgag	tggaagatga	atacttttta	acctatgtgt	atacacacat	agtaagctcg
361	Ont:	ctataatact	ttataacgtt	tttatttaca	tgagcaaagc	gagtttttcc	aacacgttta
	Olb:	1303003013	3303001233	3330333010	3202100021	2023333311	0010123330
	Glb:	1303003013	3303001233	3330333010	3202100021	2023333311	0010123330
	Gnt:	ctataatact	ttataacgtt	tttatttaca	tgagcaaagc	gagtttttcc	aacacgttta
421	Ont:	atctaaaata	ttggcaattt	ataccatgat	tttcatggta	tgtaagtgcg	cccttaggaa
	Olb:	0313000030	3322100333	0301103203	3331032230	3230023212	1113302200
	Glb:	0313000030	3322100333	0301103203	3331032230	3230023212	1113302200
	Gnt:	atctaaaata	ttggcaattt	ataccatgat	tttcatggta	tgtaagtgcg	cccttaggaa
481	Ont:	aataatttga	atatatttca	gattttcaat	ctgactgctc	ctgtcatcga	gcagaccgat
	Olb:	0030033320	0303033310	2033331003	1320132131	1323103120	2102011203
	Glb:	0030033320	0303033310	2033331003	1320132131	1323103120	2102011203
	Gnt:	aataatttga	atatatttca	gattttcaat	ctgactgctc	ctgtcatcga	gcagaccgat
541	Ont:	gaggaaaaca	aaaagaggac	taaacaaaaa	agtttagtcc	tctttttgtt	ttgaatagtt
	Olb:	2022000010	0000202201	3000100000	0233302311	3133333233	3320030233
	Glb:	2022000010	0000202201	3000100000	0233302311	3133333233	3320030233
	Gnt:	gaggaaaaca	aaaagaggac	taaacaaaaa	agtttagtcc	tctttttgtt	ttgaatagtt
601	Ont:	ctagaacgtc	atattttgcg	ttttaagcaa	ttttgactaa	ctaggcgggg	atttttactt
	Olb:	1302001231	0303333212	3333002100	3333201300	1302212222	0333330133
	Glb:	1302001231	0303333212	3333002100	3333201300	1302212222	0333330133
	Gnt:	ctagaacgtc	atattttgcg	ttttaagcaa	ttttgactaa	ctaggcgggg	atttttactt
661	Ont:	agaaattatt	caaaacgtct	gtaaagtgct	taaaatcgtt	tctaagagct	tttagcgttt
	Olb:	0200033033	1000012313	2300023213	3000031233	3130020213	3330212333
	Glb:	0200033033	1000012313	2300023213	3000031233	3130020213	3330212333
	Gnt:	agaaattatt	caaaacgtct	gtaaagtgct	taaaatcgtt	tctaagagct	tttagcgttt
721	Ont:	atttcgttta	gttatcggca	taatcgttaa	aacaggcgtt	atcgtagcgg	aaaagccctt
	Olb:	0333123330	2330312210	3003123300	0010221233	0312302122	0000211133
	Glb:	0333123330	2330312210	3003123300	0010221233	0312302122	0000211133
	Gnt:	atttcgttta	gttatcggca	taatcgttaa	aacaggcgtt	atcgtagcgg	aaaagccctt
781	Ont:	gagcgtagcg	tggctttgca	gtgaagatgt	tgtctgttag	attatgaaag	ccgataactg
	Olb:	2021230212	3221333210	2320020323	3231323302	0330320002	1120300132
	Glb:	2021230212	3221333210	2320020323	3231323302	0330320002	1120300132
	Gnt:	gagcgtagcg	tggctttgca	gtgaagatgt	tgtctgttag	attatgaaag	ccgataactg
841	Ont:	aatgaaataa	taagcgtagc	gccccttatt	tcggtcggag	gaggctcaag	ggagtttgag
	Olb:	0032000300	3002123021	2111133033	3122312202	2022131002	2202333202
	Glb:	0032000300	3002123021	2111133033	3122312202	2022131002	2202333202
	Gnt:	aatgaaataa	taagcgtagc	gccccttatt	tcggtcggag	gaggctcaag	ggagtttgag
901	Ont:	ggaatgaaat	tccctcatgg	ttttaaaatt	gcttgcaatt	ttgccgagcg	gtagcgctgg
	Olb:	2200320003	3111310322	3333000033	2133210033	3321120212	2302121322
	Glb:	2200320003	3111310322	3333000033	2133210033	3321120212	2302121322
	Gnt:	ggaatgaaat	tccctcatgg	ttttaaaatt	gcttgcaatt	ttgccgagcg	gtagcgctgg
961	Ont:	aaaatttttg	aaaaaaattt	ggaatttgga	aaaatggggg	ggtactacga	ccccccccta
	Olb:	0000333332	0000000333	2200333220	0000322222	2230130120	1111111130
	Glb:	0000333332	0000000333	2200333220	0000322222	2230130120	1111111130
	Gnt:	aaaatttttg	aaaaaaattt	ggaatttgga	aaaatggggg	ggtactacga	ccccccccta
1021	Ont:	tgtggtaatt	tggtaacttg	gtcaaaattg	atactaatat	atattaaaac	agcacaaaac
	Olb:	3232230033	3223001332	2310000332	0301300303	0303300001	0210100001
	Glb:	3232230033	3223001332	2310000332	0301300303	0303300001	0210100001
	Gnt:	tgtggtaatt	tggtaacttg	gtcaaaattg	atactaatat	atattaaaac	agcacaaaac
1081	Ont:	agaatcttat	gatataataa	gatatactga	aatttgaagg	agtaaaaaat	ggcagaagag
	Olb:	0200313303	2030300300	2030301320	0033320022	0230000003	2210200202
	Glb:	0200313303	2030300300	2030301320	0033320022	0230000003	2210200202
	Gnt:	agaatcttat	gatataataa	gatatactga	aatttgaagg	agtaaaaaat	ggcagaagag
1141	Ont:	aaaaaaagag	ttttgctaac	tttgtcgttg	gacaaagcag	aagaattaga	aactatatca
	Olb:	0000000202	3333213001	3332312332	2010002102	0020033020	0013030310
	Glb:	0000000202	3333213001	3332312332	2010002102	0020033020	0013030310
	Gnt:	aaaaaaagag	ttttgctaac	tttgtcgttg	gacaaagcag	aagaattaga	aactatatca
1201	Ont:	aaagaaatgg	gaattagtaa	atctgctctt	gttagtttat	ggattgcgga	aaattctaga
	Olb:	0002000322	2003302300	0313213133	2330233303	2203321220	0003313020
	Glb:	0002000322	2003302300	0313213133	2330233303	2203321220	0003313020
	Gnt:	aaagaaatgg	gaattagtaa	atctgctctt	gttagtttat	ggattgcgga	aaattctaga
1261	Ont:	aaataaaaaa	agagccacgg	cgaatggctc	tagtatattt	acggttagga	atattatagc
	Olb:	0003000000	0202110122	1200322131	3023030333	0122330220	0303303021
	Glb:	0003000000	0202110122	1200322131	3023030333	0122330220	0303303021
	Gnt:	aaataaaaaa	agagccacgg	cgaatggctc	tagtatattt	acggttagga	atattatagc
1321	Ont:	atatgacaga	aaaaaaacta	gaaaaaaatg	acccagttag	aaactggagt	tgggttgttt
	Olb:	0303201020	0000000130	2000000032	0111023302	0001322023	3222332333
	Glb:	0303201020	0000000130	2000000032	0111023302	0001322023	3222332333
	Gnt:	atatgacaga	aaaaaaacta	gaaaaaaatg	acccagttag	aaactggagt	tgggttgttt
1381	Ont:	atccagagtc	tgctcctgaa	aattggagaa	cattgttaga	cgaaactgga	gaaaaatgga
	Olb:	0311020231	3213113200	0033220200	1033233020	1200013220	2000003220
	Glb:	0311020231	3213113200	0033220200	1033233020	1200013220	2000003220
	Gnt:	atccagagtc	tgctcctgaa	aattggagaa	cattgttaga	cgaaactgga	gaaaaatgga
1441	Ont:	ttgagagtcc	gttgcatgat	aaagatatta	acgaaacaac	aaacgaaccg	aaaaaggcac
	Olb:	3320202311	2332103203	0002030330	0120001001	0001200112	0000022101
	Glb:	3320202311	2332103203	0002030330	0120001001	0001200112	0000022101
	Gnt:	ttgagagtcc	gttgcatgat	aaagatatta	acgaaacaac	aaacgaaccg	aaaaaggcac
1501	Ont:	attggcatat	aataatttct	ttttcaaata	aaaaaagtta	taagcaagta	ttaaaaattt
	Olb:	0332210303	0030033313	3333100030	0000002330	3002100230	3300000333
	Glb:	0332210303	0030033313	3333100030	0000002330	3002101230	3300000333
	Gnt:	attggcatat	aataatttct	ttttcaaata	aaaaaagtta	taagcacgta	ttaaaaattt
1561	Ont:	ctgaaatgtt	aaatgcacca	gagcctgtaa	aaacaaaaaa	tttacaaggg	tcagttcaat
	Olb:	1320003233	0003210110	2021132300	0001000000	3330100222	3102331003
	Glb:	1320003233	0003210110	2021132300	0001000000	3330100222	3102331003
	Gnt:	ctgaaatgtt	aaatgcacca	gagcctgtaa	aaacaaaaaa	tttacaaggg	tcagttcaat
1621	Ont:	atttgtggca	cagaaacaat	cctgaaaaat	atcagtataa	taaaagcgat	gttgttgctc
	Olb:	0333232210	1020001003	1132000003	0310230300	3000021203	2332332131
	Glb:	0333232210	1020001003	1132000003	0310230300	3000021203	2332332131
	Gnt:	atttgtggca	cagaaacaat	cctgaaaaat	atcagtataa	taaaagcgat	gttgttgctc
1681	Ont:	ataatgggtt	taaatataga	caatatttaa	cagatattgg	agttgatact	gattctattt
	Olb:	0300322233	3000303020	1003033300	1020303322	0233203013	2033130333
	Glb:	0300322233	3000303020	1003033300	1020303322	0233203013	2033130333
	Gnt:	ataatgggtt	taaatataga	caatatttaa	cagatattgg	agttgatact	gattctattt
1741	Ont:	tacaagaagt	tatagaatgg	ataaaagaaa	ctggatgttc	tgaatataga	gatttagtcg
	Olb:	3010020023	3030200322	0300002000	1322032331	3200303020	2033302312
	Glb:	3010020023	3030200322	0300002000	1322032331	3200303020	2033302312
	Gnt:	tacaagaagt	tatagaatgg	ataaaagaaa	ctggatgttc	tgaatataga	gatttagtcg
1801	Ont:	attatgcagt	atcagaacgt	ttcgatgatt	ggtttcctac	agtcagaagt	caaaccatat
	Olb:	0330321023	0310200123	3312032033	2233311301	0231020023	1000110303
	Glb:	0330321023	0310200123	3312032033	2233311301	0231020023	1000110303
	Gnt:	attatgcagt	atcagaacgt	ttcgatgatt	ggtttcctac	agtcagaagt	caaaccatat
1861	Ont:	ttttaaattc	ttatttacgc	tcaaatcgtc	atagtcagaa	aaaatataat	ccagaaacag
	Olb:	3333000331	3303330121	3100031231	0302310200	0000303003	1102000102
	Glb:	3333000331	3303330121	3100031231	0302310200	0000303003	1102000102
	Gnt:	ttttaaattc	ttatttacgc	tcaaatcgtc	atagtcagaa	aaaatataat	ccagaaacag
1921	Ont:	gagaggtgtt	atgaaagttg	aaattatagc	tagtgttttt	agtgaaaaat	cagttcagaa
	Olb:	2020223233	0320002332	0003303021	3023233333	0232000003	1023310200
	Glb:	2020223233	0320002332	0003303021	3023233333	0232000003	1023310200
	Gnt:	gagaggtgtt	atgaaagttg	aaattatagc	tagtgttttt	agtgaaaaat	cagttcagaa
1981	Ont:	aaaagtaaat	aattttattg	attatttaaa	tgacaataat	tttgaagtat	tggaagttca
	Olb:	0000230003	0033330332	0330333000	3201003003	3332002303	3220023310
	Glb:	0000230003	0033330332	0330333000	3201003003	3332002303	3220023310
	Gnt:	aaaagtaaat	aattttattg	attatttaaa	tgacaataat	tttgaagtat	tggaagttca
2041	Ont:	atatagg					
	Olb:	0303022					
	Glb:	0303022					
	Gnt:	atatagg					

The fact that a DNA sequence is identified as a sequence belonging to a codeword set of a BCH code with the minimum distance 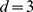 (and no other minimum distance) implies that this 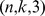 BCH code is equivalent to the Hamming code with parameters 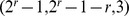, independently of the algebraic structure associated with the alphabet of the code. Therefore, the Hamming codes constructed by considering the group of units 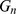 in 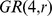 are able to identify and reproduce the DNA sequences that differ by one nucleotide from the posted *NCBI* sequences. We have also noted that the labeling, which is the set consisting of the twenty-four permutations, is split into three subsets, each of which contains eight permutations and defines a labeling denoted by 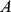, 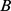, and 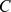 - Figure 1.

The TRAV7 predicted gene has 511 nucleotides, and therefore the codeword length is 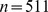 - Table 1. Using the equality 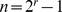, it is easy to calculate the degree 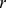 of the Galois ring extension, which is 9. The number of 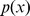 for this extension is 48 [11], [12]. Among these, just one 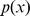 is associated with a 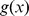 of the Hamming code (511, 502, 3), that is,
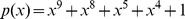
and
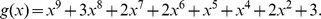
Furthermore, this identification was made using the 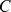 labeling.

A statistical analysis related to the *TRAV7 gene sequence* chromosome 14 of the human genome is as follows: with each primitive polynomial there is a corresponding generator polynomial of a code. For the given DNA sequence we use the 24 labeling and the resulting 24 sequences are multiplied by the generator matrix. From this operation results 24 codewords. Each one of these codewords is multiplied by the parity-check matrix. If the result is zero then the given DNA sequence is a codeword. Otherwise, we have to verify what happens if in each position we have different nucleotides. To do that, we have to realize three substitutions in each position of the original DNA sequence and verify again if this modified sequence is or is not a codeword. Since the *TRAV7 gene* genomic sequence has 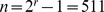, it follows that 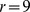. From this, the degree of the primitive polynomial is 9 and as a result we have 48 different primitive polynomials. Since for each one of them we have to use the 24 labeling, this leads to 1152 codewords to verify for a given error-correcting capability. Since in this case we have 256 possibilities, an upperbound is 294,912 codewords to be tested. Now, since there is always one nucleotide difference, we have to realize three times 63 tests for each one of the 294,912 codewords. Therefore, yielding a total of 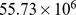 tests to be realized. Thus, the probability of finding a given sequence is 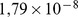, that is, approximately 1 sequence out of 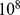.

The *Lactococcus lactis* plasmid genomic sequence has 2047 nucleotides. So, the codeword length is 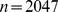 and the degree of the Galois ring extension 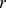 is 11. The number of 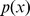 is 176 [11], [12]. Again, among these, only one 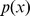 is associated with a 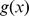 of the Hamming code 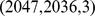, that is,
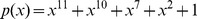
and
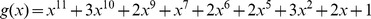
and this identification was made using the 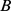 labeling, as shown in Table 2.

A statistical analysis related to the *Lactococcus lactis* plasmid genomic sequence is as follows: with each primitive polynomial there is a corresponding generator polynomial of a code. For the given DNA sequence we use the 24 labeling and the resulting 24 sequences are multiplied by the generator matrix. From this operation results 24 codewords. Each one of these codewords is multiplied by the parity-check matrix. If the result is zero then the given DNA sequence is a codeword. Otherwise, we have to verify what happens if in each position we have different nucleotides. To do that, we have to realize three substitutions in each position of the original DNA sequence and verify again if this modified sequence is or is not a codeword. Since the *Lactococcus lactis* plasmid genomic sequence has 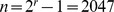, it follows that 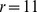. From this, the degree of the primitive polynomial is 11 and as a result we have 176 different primitive polynomials. Since for each one of them we have to use the 24 labeling, this leads to 4224 codewords to verify for a given error-correcting capability. Since in this case we have 1018 possibilities, an upperbound is 4,300,032 codewords to be tested. Now, since there is always one nucleotide difference, we have to realize three times 63 tests for each one of the 4,300,032 codewords. Therefore, yielding a total of 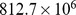 tests to be realized. Thus, the probability of finding a given sequence is 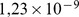, that is, approximately 1 sequence out of 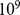.

Note that 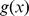 is also a primitive polynomial, since by reducing modulo 2 its coefficients leads to 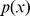. Therefore, both polynomials are associated with the same algebraic and geometric properties. Contrary to our expectations, there is just one 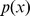, its corresponding 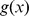, and a labeling capable of identifying each sequence under consideration. This suggests the existence of an intrinsic geometric property that may be associated with each DNA sequence.

What has been observed is that, in all the DNA sequences previously identified, there is always a difference of a single nucleotide between the *NCBI* sequence and the codeword generated by a Hamming code. Although the code (owing to its error correction capability) allows a difference in any position in the sequence, this difference occurs at one specific position. In the biological context, this mismatch is known as a single nucleotide polymorphism (SNP).

**Figure 2:**
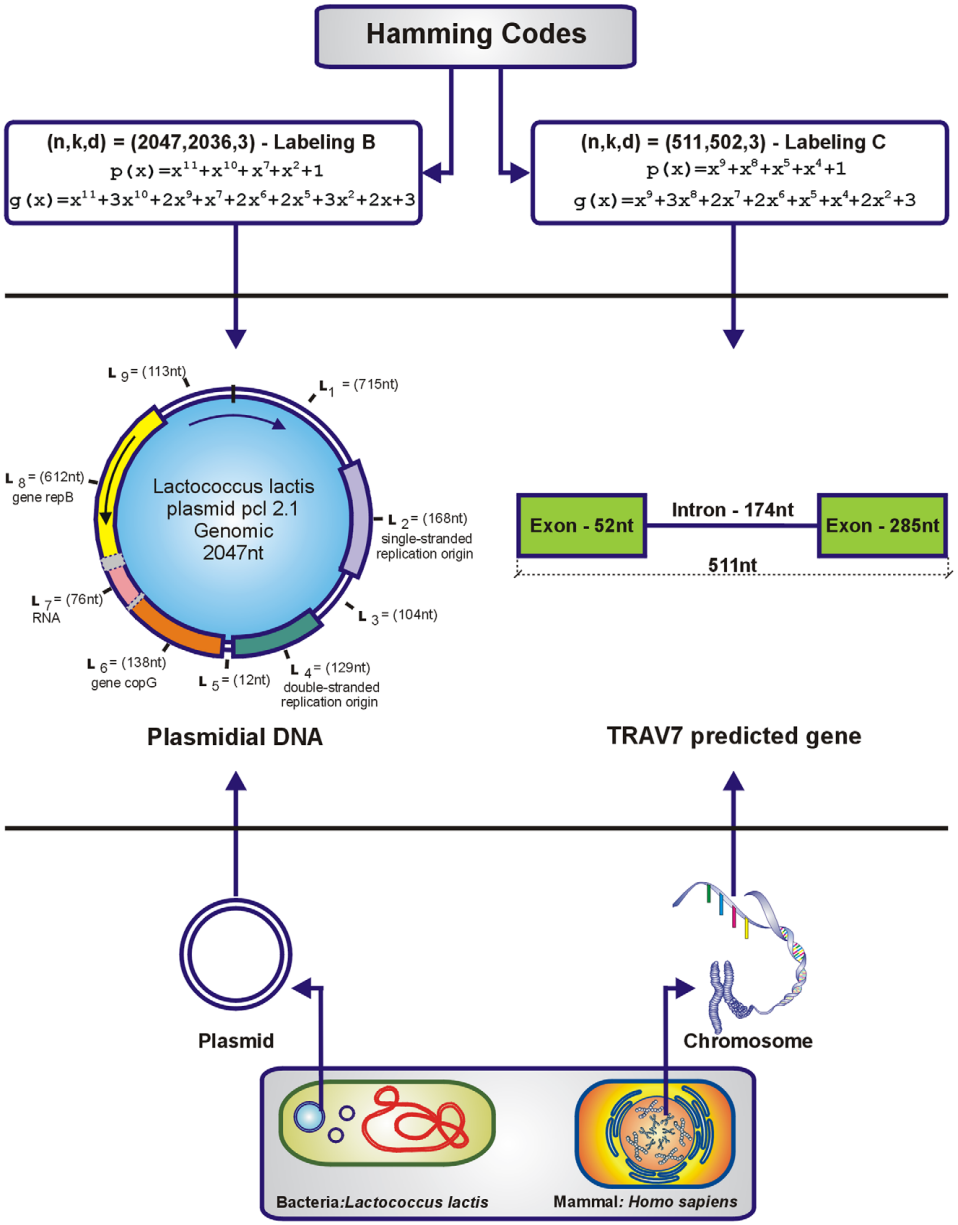
Plasmidial DNA and TRAV7 gene generation by Hamming codes.

We can observe that the SNP occurred at position 122 in the TRAV7 predicted gene, changing 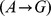, and so originating a transition mutation (change of one purine/purine or pyrimidine/pyrimidine) - Table 1. In contrast, in the *Lactococcus lactis* plasmid genomic sequence, the SNP occurred at position 1547, changing 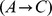, and so originating a transversion mutation (change of a purine for a pyrimidine, or vice-versa) - Table 2. Note that in the TRAV7 predicted gene the SNP occurred in the intronic region, whereas in the *Lactococcus lactis* plasmid genomic sequence the SNP occurred in the 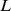 region, where the repB gene is located - Figure 2. One possible interpretation is that either the codeword generated by a Hamming code is an ancestor of the corresponding *NCBI* sequence, or it is an SNP with respect to the corresponding *NCBI* sequence, or the other way around. However, since this mismatch is within the error correction capability of the code, it follows that the modified Berlekamp-Massey decoding algorithm [15] is capable of detecting and correcting such a mismatch.

### Conclusion

In this paper, we have shown that not only are some protein coding sequences identified with the codewords of Hamming codes, but a gene, and even a whole genome, is identified with codewords as well. Although this is not a definitive answer to the question of whether or not there is an error-correcting code underlying actual DNA sequences, it is an encouraging result.

The majority of the DNA sequences were reproduced by the Hamming codes over rings. One possible explanation is provided by the arithmetic and computational flexibilities of this algebraic structure. As a consequence, sequences reproduced by the Hamming codes over fields exhibit less adaptability than those offered by the Hamming codes over rings. This observation suggests that it is possible to classify the proteins according to their stability in the mutation index.

As usually occurs when a new result appears, many new questions emerge. Do they, in fact, reveal the existence of a mathematical structure underlying DNA sequences? Why does the code point to a specific position for each reproduced sequence? Biologically, how important is the SNP in the position pointed out by the code?
