# Correction: Is a Genome a Codeword of an Error-Correcting Code?

**DOI:** 10.1371/journal.pone.0105396

**Published:** 2014-08-07

**Authors:** The PLOS ONE Staff

The statistical values are incorrect in the Results and Discussion section. The last four sentences of the fourth paragraph should read:

Since in this case we have 255 possibilities, an upperbound is 293,760 codewords to be tested. Now, since there is always one nucleotide difference, we have to realize three times 511 tests for each one of the 293,760 codewords. Therefore, yielding a total of 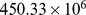 test to be realized. Thus, the probability of finding a given sequence is 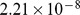, that is, approximately 1 sequence out of 10^9^.

The second equation in the fifth paragraph should be:
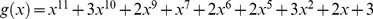



The last four sentences of the sixth paragraph should read:

Since in this case we have 1023 possibilities, an upperbound is 4,321,152 codewords to be tested. Now, since there is always one nucleotide difference, we have to realize three times 2047 tests for each one of the 4,321,152 codewords. Therefore, yielding a total of 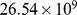 tests to be realized. Thus, the probability of finding a given sequence is 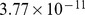, that is, approximately 1 sequence out of 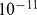.
